# Three-Dimensional Ultrasound Findings in Cornelia de Lange Syndrome: A Case Report

**DOI:** 10.1155/2012/568351

**Published:** 2012-10-08

**Authors:** Yoichiro Akahori, Hisashi Masuyama, Yumi Masumoto, Yuji Hiramatsu

**Affiliations:** Department of Obstetrics and Gynecology, Okayama University Graduate School of Medicine, Dentistry and Pharmaceutical Sciences, Okayama 700-8558, Japan

## Abstract

*Introduction*. The objective is to report a case of Cornelia de Lange syndrome (CdLS) diagnosed by detailed observations using three-dimensional sonography. *Case Report*. A 33-year-old healthy multipara was referred to our hospital at 34-week gestation after severe fetal growth restriction, congenital heart anomaly, and antebrachium abnormality were diagnosed during the third trimester. Further sonography diagnosis on cardiac abnormalities diagnosed the existence of ventricular septal defect in the outflow tract, atrioventricularis communis, and truncus arteriosus communis where the pulmonary artery branched from the common arterial trunk. As for abnormalities of the forearms, ectrodactylia and monodactylism were suspected and the abnormalities were observed sterically by using three-dimensional sonography. A 1986 g (1.07 percentile) male newborn was delivered by assisted breech extraction at 37-week gestation. After birth, from characteristic facies including bushy eyebrow, broad nasal bridge, micrognathia, and abnormalities of the forearms (ectrodactylia and monodactylism), the case was diagnosed with CdLS. *Conclusion*. Through detailed observation including abnormalities of fingers, we could exemplify this very rare disease as an antenatal diagnoses for fetal growth retardation.

## 1. Introduction

Cornelia de Lange syndrome (CdLS) is a rare fetal malformation characterized by severe fetal growth arrest, specific facial features, and major malformations (particularly the cardiac, gastrointestinal, and musculoskeletal systems). It has been estimated to occur in about 1 : 10,000 individuals but, as more mildly affected individuals have been reported, its actual prevalence may be much more [1–3].

This paper will report a case where Cornelia de Lange syndrome was suspected due to a diagnosis of fetal growth restriction, abnormalities of upper arms, and congenital heart diseases by detailed observations using three-dimensional sonography, and Cornelia de Lange syndrome was diagnosed with characteristic facies after birth.

## 2. Case Presentation

A 33-year-old healthy multipara, with no family history of Cornelia de Lange syndrome or exposure to teratogenic drugs, was referred to our hospital at 34-week gestation after severe fetal growth restriction (FGR), congenital heart anomaly, and antebrachium abnormality were diagnosed during the third trimester. In our hospital, fetal anomaly was examined in detail, using two-dimensional (2D) and three-dimensional (3D) ultrasonography (Voluson E8, GE health care Japan Co., Ltd., Tokyo, Japan). Fetal growth was restricted and type of FGR was symmetric FGR.

**Figure 1:**
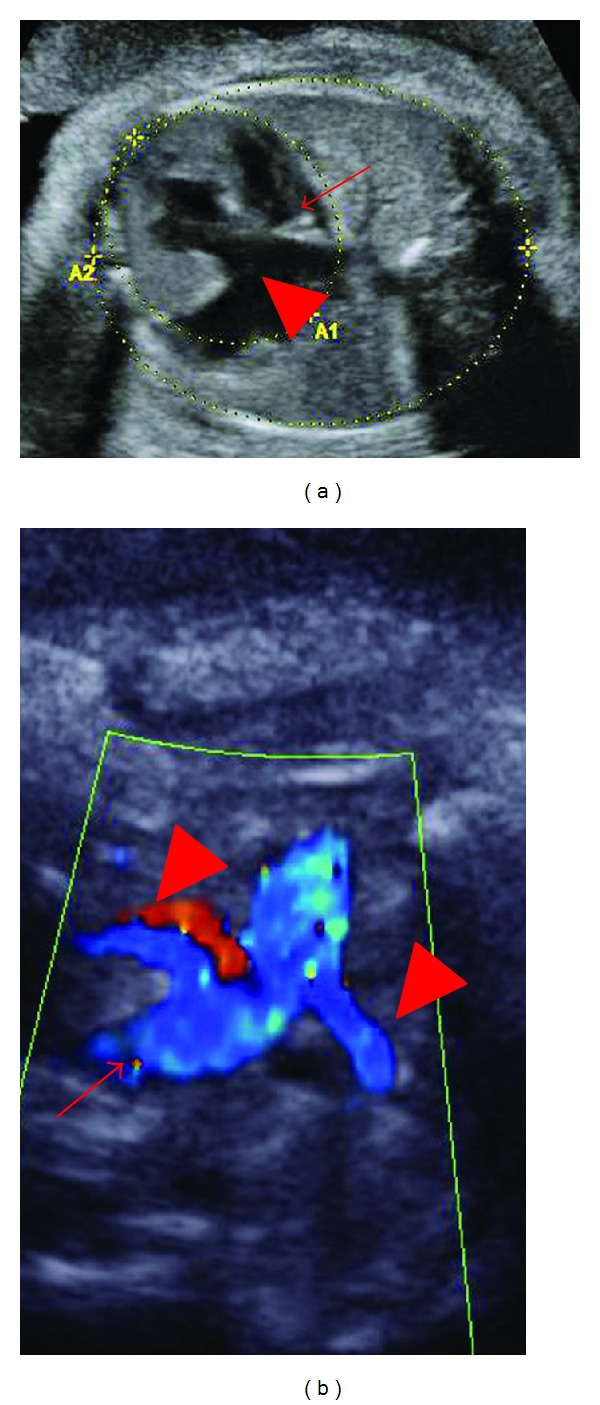
Color Doppler findings of fetal heart anomaly. (a) Ventricular septal defect (arrow):  atrioventricularis communis (arrow head), (b) truncus arteriosus communis where the pulmonary artery branched from the common arterial trunk: truncus arteriosus communis (arrow), pulmonary artery (arrow head).

**Figure 2:**
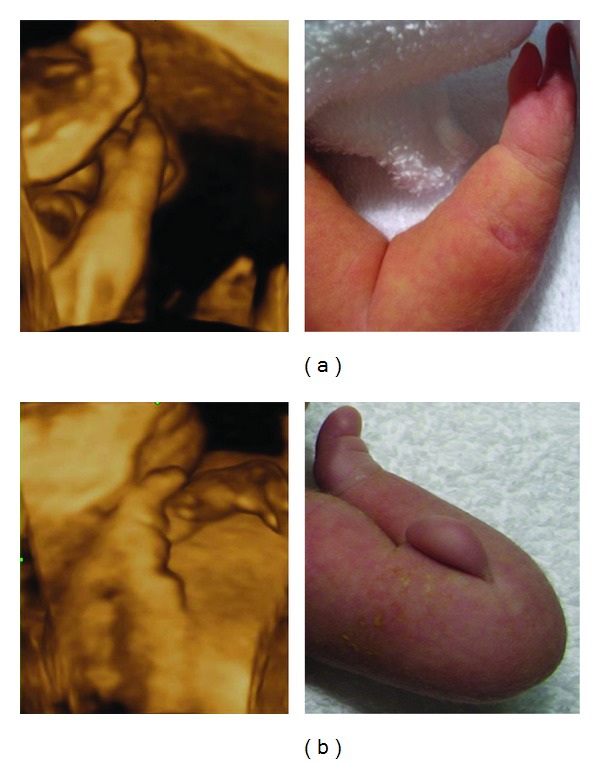
Abnormalities of the forearms in 3D ultrasonography and newborn. (a) Ectrodactylia and (b) monodactylism.

Further sonography diagnosis on cardiac abnormalities diagnosed the existence of ventricular septal defect in the outflow tract, atrioventricularis communis, and truncus arteriosus communis where the pulmonary artery branched from the common arterial trunk (Figure 1). As for abnormalities of the forearms, ectrodactylia and monodactylism were suspected (Figure 2), and the abnormalities were observed sterically by using three-dimensional sonography. Fetal karyotype analysis by amniocentesis revealed a normal karyotype of 46, XY. Viral infections during pregnancy were excluded by antibody screening. No other antenatal problems occurred, and at 37-week gestation spontaneous labor was started. 

A 1986 g (1.07 percentile) male newborn was delivered by assisted breech extraction. Apgar score was 4 and 8 at 1 and 5 minutes, respectively. After birth, from characteristic facies including bushy eyebrow, broad nasal bridge, micrognathia, and abnormalities of the forearms (ectrodactylia and monodactylism, Figure 2), the case was diagnosed with Cornelia de Lange syndrome (CdLS). Cardiac abnormalities were truncus arteriosus communis and atrioventricularis communis as in the antenatal diagnosis. On the forearms, ectrodactylia where the right two fingers were lost and monodactylism on one left finger were observed as in images taken using three-dimensional sonography. After birth, we followed up the case while orally administrating emulgent, but no apparent cardiac incompetence symptoms appeared. The patient of this case was discharged on day 48 to wait for surgery.

## 3. Discussion

CdLS is first described in 1933 and is congenital disorder characterized by growth and mental retardation and malformations of the cranial, cardiac, gastrointestinal, and skeletal systems [4]. Currently, there is no single criterion that is diagnostic for CdLS, and misdiagnosis is not common. The diagnosis, based solely on clinical assessment and recognition of the specific characteristics of this syndrome, is dependent on the identification of the distinctive facial features. Typical phenotypic findings include hirsutism, synophrys, long eyelashes, microcephaly, anteverted nostrils, prominent philtrum, and variable abnormalities of upper limbs.

It is not unusual in CdLS for congenital heart diseases (ventricular or atrial septal defects, aortic or pulmonary stenoses, tetralogy of Fallot, atrioventricular canal, single ventricle, and aortopulmonary window) to be associated with each other. In many cases of CdLS, the antenatal diagnosis is made after postnatal evaluation [5]. After detailed evaluation of the fetal heart, this case was able to be diagnosed with truncus arteriosus communis. As the association of cardiac abnormalities expects life prognosis of CdLS, antenatal diagnosis of cardiac abnormalities is especially important.

There are several reports on antenatal diagnosis of CdLS [5–9]. By using intrauterine growth retardation as a trigger for antenatal diagnosis, CdLS is diagnosed by abnormalities of upper arms, congenital heart diseases, and characteristic facies. Recently, with the advancement of medical sonography equipment, antenatal diagnosis can be conducted more sterically using three-dimensional sonography. The past records also described the effectiveness of three-dimensional sonography for antenatal diagnosis of CdLS [8–10]. In this case, intrauterine growth retardation triggered detailed examinations; facial abnormalities and ectrodactylia and monodactylism were sterically observed using three-dimensional sonography, which was effective for antenatal diagnosis of CdLS. As CdLS is accompanied by facial and upper arm abnormalities, three-dimensional sonography which observes a fetus sterically could be effective for antenatal diagnosis.

We experienced one case of Cornelia de Lange syndrome. Through detailed observation including abnormalities of fingers, we could exemplify this very rare disease as an antenatal diagnosis for intrauterine growth retardation.
